# Student nurses’ perspective on readiness for clinical practice post-COVID-19 in South Africa

**DOI:** 10.4102/hsag.v30i0.2790

**Published:** 2025-03-05

**Authors:** Kelebogile P. Olyn, Tennyson Mgutshini

**Affiliations:** 1 Department of Health Studies, College of Human Sciences, University of South Africa, Pretoria, South Africa

**Keywords:** adaptation, intimidation, open-mindedness, perspectives, post-COVID-19, readiness to clinical practice, strained relationships, student nurses

## Abstract

**Background:**

The clinical learning environment (CLE) provides student nurses with practical experience and skill development. However, COVID-19 restrictions have raised concerns about their readiness for clinical practice.

**Aim:**

This study examined student nurses’ readiness for clinical practice post-COVID-19 at two nursing institutions in South Africa.

**Setting:**

The study was conducted with student nurses from two selected nursing education institutions in two South African provinces.

**Methods:**

A qualitative, explorative, and descriptive approach was used to gather perspectives from 3rd- and 4th-year student nurses. Two focus groups were conducted at each institution, achieving data saturation with 31 participants. The data was audio recorded with their consent. Using Braun and Clarke’s framework, descriptive thematic analysis was employed.

**Results:**

The study identified two main themes: (1) Positive perspectives: Adaptation and Open-mindedness, and (2) Negative perspectives: Intimidation and Strained Relationships.

**Conclusion:**

Participants emphasised the necessity for adaptation and innovation during the pandemic. Although intimidation and strained relationships impacted their confidence, these challenges also promoted personal growth and development. Student nurses demonstrated significant adaptability and openness to innovation, which enhanced their learning and readiness for clinical practice (RtCP) post-COVID-19.

**Contribution:**

Despite facing intimidation and strained relationships, these experiences fostered both personal and professional growth, improving employability. The study underscores the critical role of adaptability and innovation in nursing education, particularly in the post-COVID-19 context. Mentorship and supportive environments can mitigate intimidation and strained relationships, thereby boosting confidence and autonomy, and resulting in more competent nursing professionals.

## Introduction

Readiness to the clinical practice (RtCP) encompasses the student nurses’ (STNs) capacity to secure and sustain initial employment, comprehend the essential competencies within the clinical learning environment (CLE) and engage effectively within an integrated educational system (O’Keefe & Auffermann 2022). This process involves the STNs’ ability to transition between various roles and positions within a CLE, aligning with organisational demands and effectively managing shared skills across institutions. By continuously generating work through optimal effort utilisation, STNs can enhance their adaptability and open-mindedness (Liu et al. 2021).

In this study, readiness denotes the preparedness of STNs to engage in clinical practice, highlighting adept interpersonal relationship skills (IPRS) after their designated training period.

Triemstra et al. (2021) highlight the coronavirus disease 2019 (COVID-19) pandemic as a significant 21st-century crisis, profoundly impacting global nursing training both physically and psychologically. Vulnerable groups, including STNs, face unique challenges. The pandemic’s stress, anger and uncertainties, along with lockdown measures, have placed STNs in a demanding CLE, exposing them to various patient and infection risks (Bowser et al. 2022).

The fear of contracting the virus adds stress to STNs, potentially hindering the open-mindedness and adaptability needed for effective practice in CLE (Liu et al. 2021). Thus, fostering these qualities was crucial as STNs navigated patient care complexities during COVID-19, enabling them to adapt to changing circumstances, embrace diverse perspectives and meet the dynamic demands of their roles post-pandemic (Bowser et al. 2022).

Castro et al. (2021) stress the need for STNs to adapt to healthcare changes and global shifts to enhance services. Hanson, Kim and Badowski (2023) note that neglecting IPRS in nursing can hinder effective teams in CLE, affecting patient care and collaboration. Thus, STNs must quickly adapt to the post-COVID-19 CLE by developing IPRS and gaining new knowledge.

The literature emphasises that adaptability and open-mindedness are key for balancing academics, clinical placements and personal life, helping prevent burnout through strong interpersonal skills (Castro et al. 2021; Liu et al. 2021). Bowser et al. (2022) emphasise that patients’ perceptions of STNs interactions shape their care experience. Positive interactions foster collaboration, making adaptability and open-mindedness in STNs crucial for better patient outcomes and healthcare quality.

Huang and Fang (2023) emphasise that fostering open-mindedness in nursing education relies on professional behaviour, ethical standards and expertise. Developing adaptability and open-mindedness is crucial for building reliable relationships, especially during challenges like the COVID-19 pandemic. Student nurses also need strong advocacy skills to connect with patients and appeal to employers seeking patient-centred care (Bowser et al. 2022). This requires an open-minded approach to understanding diverse patient needs and perspectives. By being adaptable, STNs can tailor their advocacy efforts to effectively address the unique circumstances of each patient, ensuring that care is both personalised and comprehensive. Open-mindedness and adaptability are essential in navigating the dynamic healthcare environment and fostering meaningful connections with both patients and employers.

Kaveh et al. (2022) found that discussions among nursing staff are beneficial when both professional and STNs are open to new and established insights. This openness enhances collaboration and cohesion, leading to better student outcomes and approaches to future challenges. In the CLE, respectful listening to all perspectives ensures meaningful outcomes.

Kaveh et al. (2022) advocate for educators to adopt an open-minded approach in applying theoretical knowledge to practice, emphasising flexibility in expressing fears and sharing experiences. This promotes open-mindedness in student development for RtCP though the study did not address its impact on patient outcomes.

Jung et al. (2022) found significant correlations between STNs’ personality traits, attitudes, acceptance of open-mindedness, adaptability and new learning situations. In a qualitative study Younis, Anwar and Batool (2024), concluded that open-mindedness’ effectiveness is based on behaviour and attitude, influenced by norms and free will, allowing nurses to control their growth and resist others’ actions.

Kaveh et al. (2022) suggest that open-minded nurse educators enhance STNs’ development by embracing new perspectives, improving critical thinking, social skills and emotional stability, which is crucial for RtCP. The study concludes that open-mindedness in nursing education leads to higher quality care and a sense of security for STNs. However, adaptability is also essential for clinical readiness (Bongelli et al. 2021). Jung et al. (2022) argue that open-mindedness and adaptability in CLE readiness stem from personal experiences and exposure. Post-COVID-19, nursing education should prioritise fostering open-mindedness and teamwork through active engagement with STNs.

Wang et al. (2022) found that skill transfer among STNs, driven by peers and mentors, involves role-modelling flexibility skills. This approach boosts STNs’ confidence and open-mindedness, making them feel valued. The research indicates a growing interest in enabling STNs to share their understanding of concepts freely, enhancing their RtCP.

### Problem statement

A key issue in the CLE during the COVID-19 pandemic has been the need for greater flexibility and open-mindedness among STNs. Research shows that these qualities are vital for adapting to pandemic challenges; yet, shortcomings in the CLE have hindered STNs’ preparation, revealing gaps in support (Lanahan, Montalvo & Cohn 2022). However, there is a significant gap in integrating these perspectives into nursing education. This gap is concerning given the increasing frequency of global disasters and conflicts, which requires a workforce that is both clinically competent and adaptable. Therefore, nursing education programmes must evolve to emphasise these critical attributes, ensuring future nurses are well prepared for unprecedented challenges (Lanahan et al. 2022).

Kim and Lee (2022) note that the CLE often fails to cultivate these essential skills, which are vital for adapting patient care methods and enhancing collaboration. Despite efforts to address these gaps, nursing curricula during the pandemic have continued to challenge patient care and health outcomes.

Haslam (2021) notes that STNs were overwhelmed by the pandemic’s severity, high patient volumes and intimidation by healthcare personnel. This environment hindered their ability to voice concerns and understand issues, affecting their readiness for post-COVID-19 clinical practice and progress toward Sustainable Development Goal (SDG) 3.

In South Africa, the pandemic exposed additional challenges for STNs, particularly in two provinces where poor interpersonal skills hindered their ability to keep up with clinical advancements. These deficiencies highlight the need for integrating critical thinking, adaptability and open-mindedness into nursing education. Addressing these gaps can better prepare STNs for future global health crises, ensuring they provide high-quality, patient-centred care.

This study aims to explore STNs’ perspectives on RtCP in the post-COVID-19 context, offering insights to improve nursing education and CLE. Understanding these perspectives can help educators and healthcare leaders develop strategies to enhance the preparedness and resilience of future nursing professionals.

### Theoretical framework

This study integrates the Theory of Planned Behaviour (TPB) with Attribution Theory (AT) to examine how STNs’ perspectives during clinical placements influence their RtP post-COVID-19 (Arnold & Boggs 2019). According to TPB, STNs’ positive or negative perspectives significantly impact their adaptability and open-mindedness. Attitudes influence their readiness to engage in behaviours that promote adaptation and innovation in nursing education and CLE. Consequently, STNs with positive attributes are better prepared to adapt to the evolving healthcare landscape post-pandemic (Palacios-Ceña et al. 2022).

Negative traits such as intimidation and strained relationships, often stemming from heavy workloads and safety concerns, impede RtCP. According to the TPB, these external factors affect STNs’ confidence and autonomy, diminishing their control over experiences and hindering RtCP post-COVID-19. However, these challenges can also foster personal growth and career advancement. Theory of Planned Behaviour highlights the significance of behavioural beliefs and subjective norms. Student nurses’ who prioritise adaptation and innovation are more likely to embrace these behaviours because of social pressures, thereby enhancing their readiness for dynamic healthcare environments post-pandemic (Castro et al. 2021).

Attribution theory suggests that STNs’ perceptions significantly influence their RtCP post-COVID-19 by examining how they interpret events and attribute causes. During the pandemic, STNs’ rapid adaptation and assumption of responsibilities were linked to internal factors like dedication and resilience (Drach–Zahavy et al. 2022). Chaotic conditions were attributed to external, uncontrollable factors. Despite challenges, STNs’ adaptability and open-mindedness indicate an internal locus of control, reflecting their belief in influencing outcomes. Attribution theory posits that those attributing experiences to internal factors feel empowered to continue nursing, while those attributing them to external factors may consider leaving the profession (Kim & Lee 2022).

### Research aim

The study aimed to explore and describe STNs perspectives on readiness for clinical practice in the post-COVID-19 era, exploring the impact of the pandemic on their readiness for the CLE in two selected nursing education institutions (NEIs). Additionally, the study sought to suggest recommendations to support a smooth transition for STNs in the post-pandemic era.

## Research methods and design methods

### Research design

The research utilised a qualitative, exploratory and descriptive design. By adopting a constructive paradigm, the study aimed to interpret the topic from the participants’ perspectives (Creswell 2021).

### Research setting

The study was conducted with STNs from two selected NEIs in two South African provinces. These NEIs enrol STNs in undergraduate programmes and arrange placements in various district hospitals within the provinces. Conducted in a naturalistic setting, the study focussed on STNs’ perspectives of clinical learning experiences and their RtCP post-COVID-19 (Polit & Beck 2021). The placements in different district hospitals aimed to correlate theoretical knowledge with practical experience.

### Population and sampling

A population refers to the larger group from which a sample is drawn, encompassing units, individuals, objects, systems or organisations (Polit & Beck 2021). The study included 410 third-year and 290 fourth-year STNs. The researcher purposively selected the NEIs from both urban and rural provinces to gather diverse perspectives and enhance the generalisability of the results.

Purposive sampling was employed to select STNs likely to provide valuable insights (Gray, Grove & Sutherland 2017). Four Focus Group Discussions (FGDs) were conducted, each with 7–8 participants. The researcher explained to participants about the study, distributing information leaflets and contact details. Interested STNs contacted the college research chairperson, who then forwarded their details to the researcher for scheduling.

### Data collection

Following approvals from relevant ethics and research committees, the researcher obtained written consent from the two chosen NEIs. The researcher began with laying the ground rules for each session of FGD for smooth data collection. The purpose of data collection was explained, and the participants consented to the process by signing consent forms to affirm that they were participating in the study out of their own free will. They also consented to the focus group interviews being audio recorded. The seating arrangement of the participants was in a circular pattern to allow for face-to-face engagement between the researcher and the participants. Data collection was guided by the use of an interview guide; this consisted of a major question followed by probing and follow-up questions. The aim of the FGD was to obtain rich data from the participants in their own setting. All the FGDs lasted on average, for an hour, and fieldnotes were also captured. Data collection was conducted until saturation was reached at the fourth FGD (Polit & Beck 2021).

### Data analysis

Data analysis was conducted concurrently with data collection using Braun and Clarke’s (2013) six-step framework. The researcher transcribed audio recordings verbatim. The steps included: (1) familiarisation with the data through re-reading transcripts and field notes, (2) generation of initial codes to collate data, (3) identification of patterns by tagging themes, (4) reviewing themes by searching for relationships between repeated ideas, (5) defining and naming themes and (6) producing the final report. The analysis resulted in the identification of two themes and four sub-themes.

### Measures of trustworthiness

The data quality was ensured by adhering to Guba’s 1981 model of trustworthiness using the principles: credibility, dependability, confirmability and transferability (Krefting 1991). Prolonged engagement and adequate probing ensured credibility. Transferability was ensured by writing the field notes during the interviews and verbatim transcriptions. Dependability was ensured by probing and rephrasing unclear questions. Confirmability was ensured by using audio recordings and field notes.

### Ethical considerations

The researcher strictly adhered to the ethical guidelines established by the University of South Africa (UNISA) Research Ethics Committee. After receiving ethical clearance (REC-012714-039), further approval was secured from the Provincial Research Committee, the Provincial Research Committee of the Department of Health, and the principals of the nursing education institutions. To protect confidentiality, interview data were anonymised by replacing participants’ names with coded identifiers. Participants were fully informed about their voluntary participation and their right to withdraw from the study at any time. The research upheld fundamental ethical principles, including anonymity, autonomy, beneficence, and justice, all of which were clearly communicated and consistently maintained throughout the study.

## Results

### Demographics data

A total of 31 STNs from two provinces and NEIs took part in the FGDs (Table 1). The group was largely female, with 29 women and only 2 men, highlighting the female-dominated nature of the nursing profession. This imbalance could affect the findings, potentially overlooking important nuances from the perspectives of male nurses, who may encounter distinct experiences and challenges related to RtCP post-COVID-19. Despite their limited numbers, the male participants openly shared their views, reflecting the realistic but minimal representation of men in nursing, which limits the external validity of the findings.

**TABLE 1:** Demographics of participants.

Classification of provinces	NEI	Year of study	Gender		Ethnicity	
			Male	Female	Black	Other
Province A	NEI A	3rd	2	6	8	0
	NEI B	4th	0	8	7	1
Province B	NEI A	3rd	0	7	7	0
	NEI B	4th	0	8	8	0

**Total**	-	-	**2**	**29**	**30**	**1**

NEI, nursing education institution.

Four participants, identified as recognition of prior learning (RPL) STNs, brought extensive pre-pandemic clinical experience from two public hospitals. Their practical insights were invaluable because of their prolonged exposure to the clinical environment. They expressed their concerns, given the rare opportunity to further their studies under strict conditions, while balancing family and hospital responsibilities. Their readiness to practice was crucial, providing significant perspectives to the less experienced participants in the group discussions.

The group consisted predominantly of black participants, with only one Indian STN, reflecting the largely black local community. The distinct perspectives and cultural backgrounds of the Indian participants may not have been fully captured, leading to an incomplete understanding of the broader STN experiences. While this limited representation does not fully encompass the Indian community’s viewpoint, the qualitative interview offered valuable insights that warrant further exploration. Despite cultural differences, the participant’s contributions were instrumental in highlighting important considerations.

Two main themes emerged from data collected from focus groups: Positive and negative perspectives, each with two sub-themes as discussed in Table 2.

**TABLE 2:** Themes and sub-themes that emerged from the findings.

Themes	Sub-themes
1. Positive perspectives	1.1 Adaptability 1.2 Open-mindedness
2. Negative perspectives	2.1 Intimidation 2.2 Strained relationships

### Theme 1: Positive perspectives

From the perspective of STNs on RtCP in the post-COVID-19 era, participants saw the challenges they faced as opportunities for growth. These experiences fostered their adaptation and flexibility, enabling them to develop critical thinking skills in complex situations. They highlighted the importance of listening to others and maintaining an open-minded approach, which significantly enhanced collaboration in patient care.

#### Sub-theme 1.1: Adaptability

Flexible STNs are willing to explore diverse perspectives, modify patient care approaches and contribute to healthcare teams, enhancing their RtCP as competent professionals.

One STN said:

‘As student participants, the COVID-19 pandemic presented daunting challenges, erasing any sense of routine. I quickly adapted to the urgency, engaging in critical tasks like triaging patients and assisting the medical team. The circumstances demanded swift action and adaptability, urging us to contribute to patient care and support the healthcare team.’ (P-2, FG2, 26 years old, Female)

Another STN mentioned:

‘The situation was chaotic and demanding. We had to think critically and balance patient care with safety, utilising limited resources like masks and gloves. Witnessing patients in pain without adequate medication was challenging, but we learned to provide psychological support.’ (P-6, FG1, 25 years old, Male)

One student still showed shock on her face:

‘We faced a challenging situation where a patient went into labour, but all beds were occupied. We improvised by placing her on a stretcher, feeling uneasy due to the unconventional circumstances. Despite limited supplies and shared resources due to COVID-19, both the mother and child were saved. This experience strengthened me and provided hope, reminding me not to succumb to anxiety.’ (P-1, FG3, 28 years old, Female)

Another student spoke about the compromises they had to make for their safety during the COVID-19 pandemic:

‘… we faced a lack of educators due to the circumstances. Despite their absence, we were directed to continue our duties, relying heavily on trial and error. The shortage of personal protective equipment was unsettling, so I brought homemade masks and conserved gloves.’ (P-3, FG4, 26 years old, Female)

The STNs were compelled to collaborate and utilise available resources to maximise their performance despite the challenging circumstances. This experience underscored the significance of openness and effective listening, leading to the development of the following sub-theme.

#### Sub-theme 1.2: Open-mindedness

From the perspective of STN on RtCP in the post-COVID-19 era, open-mindedness played a crucial role in creating a supportive CLE despite uncertainties and limited resources. Initially dependent on one another, STNs gradually developed teamwork and empathy, sharing knowledge and resources to ensure quality care under constraints. This principle is essential for their future readiness, demonstrating a strong commitment to collaboration and mutual support in their professional careers.

Supporting the sub-theme, one student stated:

‘… the evolving patient care guidelines left us feeling bewildered and unsure of our responsibilities. To cope, we prioritised discussing the new protocols whenever possible. Carefully listening to each other’s different opinions rescued us. Sharing knowledge and experiences provided mutual reassurance and strengthened our resolve to confront challenges.’ (P-5, FG3, 24 years old, Female)

One student indicated that:

‘… the uncertainty extended to ward allocations, with assignments changing frequently based on immediate needs. This sometimes resulted in finding oneself in unfamiliar units without any familiar faces from our NEI. Despite this, I made a conscious effort to listen and seek assistance from anyone willing to lend a hand. Maintaining a spirit of respect and humanity proved essential in navigating these unfamiliar situations.’ (P-6, FG4, 32 years old, Female)

Another student shared the experience:

‘The pandemic heightened anxiety and stress due to daily uncertainties and workload. The fear of mortality left me feeling vulnerable. As a group, we leaned on each other for support whenever we crossed paths. The atmosphere was fraught with drama and fear, creating unease, but here we are, we survived holding hands.’ (P-7, FG1, 26 years old, Male)

One participant shared their growth experience:

‘Amidst the chaos, I recognised the value of openness to new ideas. This mindset shift exposed us to different approaches, ensuring better preparation for future pandemics. I anticipate facing anxiety with confidence, having grown from this experience.’ (P-1, FG4, 25 years old, Female)

The positive attributes displayed by STNs were overshadowed by negative aspects that left them compromised in their RTP post-COVID-19 pandemic. The next theme emerges from the contradictions experienced by the STNs, detailing how they navigated these challenges and the barriers they encountered.

### Theme 2: Negative perspectives

In this theme, the STNs reflected on the pandemic’s severity and the overwhelming number of patients, compounded by limited resources, which unnerved them. Additionally, healthcare professionals in the CLE made it difficult for them to express their fears and concerns. Student nurses identified this as a significant drawback, potentially influencing their career decisions. Two sub-themes: intimidation and restrained relationships that emerged under this theme are discussed next.

#### Sub-theme 2.1: Intimidation

The theme of intimidation emerges from the limited resources and unclear guidance provided for patient care tasks, leading to anxiety among STNs about completing their courses, especially because of suspended clinical placements and the lack of clear direction from educators. In addition, some STNs felt victimised because of their status, making them vulnerable and underrepresented. This dependency on the system for course completion instils fear during clinical placements. These are some of their voices:

‘The COVID-19 pandemic’s heavy workload and limited resources overwhelmed. Uncertainty about expectations led to urgent tasks, often standing in for sick personnel while dealing with personal health challenges. I even contemplated quitting the nursing profession, unsure if it is truly what I desire.’ (P-2, FG1, 23 years old, Female)

‘For a while, I’ve worried we wouldn’t finish our course this year. Educators expressed empathy but lacked clear solutions. I felt sorry for them when clinical placement was suspended, and we were told to stay home. It was intimidating, and we could not reach out to educators for guidance.’ (P-3, FG3, 26 years old, Female)

Another participant spoke of their feeling of despair:

‘Ma’am, our class feels marginalised and silenced. We are labelled as the “black sheep” class and struggle to voice our concerns. Some STNs had to repeat the year due to personal challenges like difficult pregnancies or spiritual issues. It is hard for us to speak up; we feel misunderstood and underrepresented, lost in translation.’ (P-4, FG1, 22 years old, Female)

With a painful facial expression, another student said:

‘As a sheepskin group, Ma’am, we are at the mercy of understanding objectives. I agree and keep quiet to avoid trouble. We write exams without books or support, but fear being victimised and failing again.’ (P-5, FG1, 30 years old, Female)

Transitioning from this theme, we now explore the theme of restrained relationships. This theme examines how the pressures and uncertainties of their CLE impact the STN’s interactions and relationships with their peers and educators.

#### Sub-theme 2.2: Strained relationships

The theme of restrained personal relationships examines how the pressures within the CLE, compounded by the COVID-19 pandemic, have strained interactions among STNs and between STNs and educators. This has resulted in a tense and divided atmosphere, impeding effective collaboration and support.

One of the participants spoke about unapproachability that leads to isolation and frustration:

‘Sometimes you can see from a distance that the lecturer today is not in a good mood, we are afraid to speak out, we do not want to fail again.’ (P-4, FG3, 26 years old, Female)

Another student talked about the sense of helplessness and a lack of support:

‘… you just come to work even when you are sick, keep quiet to avoid being hated, it was painful during COVID-19, I am sick, the patient is sick, and we are both vulnerable, but what can we do? We are afraid to fail again.’ (P-2, FG4, 28 years old, Female)

Participants were also vocal about the lack of empathy:

‘… the lecturer will say, you are an adult learner, go find out for yourself, you can see that she does not like you because you talk too much, I just do my best to assist the patients I am allocated to work with, trying to make sure they do not see what is going on, but sometimes they can pick it up and ask if one is sure of what should be done. It can be very painful.’ (P-6, FG3, 32 years old, Female)

They also commented on the intrapersonal relationship among students:

‘Fellow STNs also, mainly the young ones, talk to us older STNs as If we don’t know what we are doing, sometimes very rude, so to work in an environment that is tense, I rather move away and not talk about the matter at all, but when the patient is involved just do it and that cause friction between us.’ (P-7, FG4, 44 years old, Female)

The participants also spoke about dynamics between older and younger STNs:

‘They act like they know it all, worsening the already poor collaboration among us during COVID-19. When help is offered, they resist, creating a divide between two unsupportive groups. It is frustrating because at the end, patient care comes first even when we are all afraid to die.’ (P-5, FG2, 39 years old, Female)

## Discussion

The study sought to explore the perspectives of STNs regarding their RtCP in the post-COVID-19 era within healthcare CLEs in two provinces of choice. From the viewpoints of STNs, it becomes apparent that they encountered unprecedented challenges and uncertainties during this period.

According to STNs’ perspectives, the COVID-19 pandemic presented significant challenges in balancing clinical work with virtual classes, as highlighted by O’Keefe and Auffermann (2022). Despite these obstacles, STNs developed new strategies to adapt. The phases of confusion, withdrawal and adjustment were notable barriers; yet, the necessity to adapt to infectious disease scenarios ultimately strengthened their relationships and prepared them for future growth (Kwon et al. 2022).

Viorato-Romero et al. (2023) found that the CLE significantly influenced the preparation of STNs for post-COVID-19 clinical practice. Their research in South Africa’s Western Cape revealed that STNs perceived the pandemic transition as highly stressful and lacked adequate support. This perceived lack of support could hinder their RtCP, underscoring the importance of a supportive and adaptive CLE. Furthermore, Bowser et al. (2022) emphasised that STNs learned to rely on themselves and share skills amidst adversity, fostering critical thinking, decision-making and positive attitudes towards their career development. This self-reliance and skill-sharing were crucial in navigating the challenges posed by the pandemic, highlighting the need for nursing education programmes to incorporate these experiences into their curricula to better prepare STNs for future global health crises.

Drasiku et al. (2021) reveal that the transition of new graduate nurses to clinical practice in Uganda is influenced by factors like personal attributes, interactions and organisational dynamics, many beyond their control. Bongelli et al. (2021) emphasise the critical role of adaptation in the post-COVID-19 environment, expanding learning strategies for STNs through tools like Google Scholar and WhatsApp. The ability of STNs to make quick decisions and efficiently triage patients highlights their essential adaptability.

The shortage of educators exacerbates challenges for STNs. Drasiku et al. (2021) cite Uganda as an example of resilience, where STNs persist and use trial-and-error methods despite the lack of educator guidance. This resilience underscores the need for nursing education programmes to support STNs in developing these skills. Mazalová, Gurková and Štureková (2022) highlight the significant impact of the CLE and the attitudes of educators and clinical staff on the adaptability of STNs in the Czech Republic. Ulenaers et al. (2021) stress the importance of overcoming obstacles to deliver high-quality care, noting the scarcity of critical resources like masks and gloves among STNs, which worsens their circumstances.

Wang et al. (2022) emphasise the importance of open-mindedness for enhancing cultural immersion and promoting RtCP, aligning with the current study’s findings that highlight its role in adapting to diverse clinical environments. On the other hand, Kim and Lee (2022) analyse self-protection methods used by STNs, such as crafting homemade masks and conserving gloves, reflecting personal sacrifices and adaptability. These strategies underscore the need for STNs to develop innovative approaches to cope with resource limitations.

The latest literature suggests that neglecting open-mindedness and adaptability can lead to negative outcomes, as seen in the current study where the absence of these qualities resulted in difficulties adapting to changing circumstances and addressing dynamic demands (Luhanga et al. 2023). In Spain, Palacios-Ceña et al. (2022) concur with the findings of the present study, highlighting that ethical dilemmas and conflicts experienced by STNs contribute to the discourse on fostering adaptability and open-mindedness in situations of uncertainty. The key focus of the current study is that the CLE should be both supportive and adaptable to effectively prepare STNs for future challenges. This aligns with the current study’s emphasis on the need for a supportive and adaptive CLE to prepare STNs for future challenges (Palacios-Ceña et al. 2022).

Ulenaers et al. (2021) support this study’s findings on strained relationships, noting that STNs often feel uncertain and confused about their responsibilities because of frequent changes in patient care guidelines. This uncertainty, along with educator tension, compromises patient care and collaboration. Despite efforts to adapt, the trauma from strained relationships could threaten their RtCP and patient care. Triemstra et al. (2021) found that fostering open-mindedness among STNs promotes empathy, mutual support and collaboration, especially during crises. However, Luhanga et al. (2023) emphasise the need to address challenges in RtCP programmes that hinder solidarity and teamwork among nursing professionals. Student nurses often feel intimidated and vulnerable, particularly regarding the completion of their studies.

Literature records increased uncertainty among STNs because of unclear guidance from educators who are also unsure about navigating the current landscape (Viorato-Romero et al. 2023). The suspension of clinical placements worsens their anxiety, leaving them without proper direction. Additionally, Luhanga et al. (2023) describe the intimidation STNs face, including unfair treatment and denial of opportunities. Triemstra et al. (2021) suggest these experiences can impact STNs’ emotional intelligence and readiness for future careers. Despite these challenges, uncertainties in studies and placements may prompt STNs to reflect, identify strengths and foster personal growth, leading to resilience and adaptability. Therefore, nursing education programmes must address these issues, providing clear guidance and support to prepare STNs for the complexities of clinical practice in a post-COVID-19 world.

This study finds that during times of uncertainty and intimidation, STNs often find comfort in shared knowledge and experiences, fostering a supportive network (Viorato-Romero et al. 2023). However, the lack of support and fear of victimisation present significant challenges, especially during exams without sufficient resources (Kwon et al. 2022). Conversely, Haslam (2021) suggests that challenging exams encourage STNs to develop resourcefulness and problem-solving skills.

In South Africa, Masso et al. (2022) found external factors impacting STNs’ RtCP. These factors can inadvertently build resilience, prompting coping strategies for challenging situations. Moreover, prolonged exposure to such challenges can diminish STNs’ confidence and knowledge, leading to feelings of inadequacy in the CLE and potentially impacting their RtCP (Nodine et al. 2021). This aligns with current findings, indicating that sustained difficulties contribute to a decline in STNs’ self-assurance and competence.

## Recommendations

### To the nursing practice

Healthcare organisations should prioritise crisis response protocols, emphasising rapid decision-making and adaptability in chaotic situations like the COVID-19 pandemic. Nursing staff should receive regular training to build resilience and enhance adaptability skills. Additionally, organisations must prioritise staff well-being by providing mental health resources, debriefing sessions and support systems for burnout and compassion fatigue. Clear protocols addressing disrespectful behaviour in CLE are essential, including reporting channels, investigations and disciplinary measures for a safe learning environment.

### To the nursing education

Nursing education programmes should integrate crisis preparedness and management modules covering rapid decision-making, triage protocols and effective communication during emergencies. Enhancing hands-on training in high-stress environments is vital. Emphasising self-care and resilience-building techniques from the start ensures students learn stress management, work-life balance and seeking support. Peer support networks in nursing schools should offer encouragement and advice for coping and resilience. Encouraging self-reflection and self-care is essential for students to advocate, manage stress and prevent burnout in their education and nursing careers. This should be a mandatory stand-alone module.

### For policymakers

The policy should work on the development of programmes aimed at fostering mitigating negative impact and inclusive CLE to facilitate STNs’ engagement in learning and professional growth.

### For future research

Conduct long-term studies to track the progress and adaptation of STNs over time, assessing how initial challenges and coping strategies influence their professional development and readiness for clinical practice.

Investigate the effectiveness of specific intervention programmes designed to enhance open-mindedness, adaptability and resilience among STNs. This could include mentorship programmes, stress management workshops and simulation-based training.

Compare the experiences of STNs in different regions or countries to identify common challenges and successful strategies. This can help in developing globally applicable best practices for nursing education.

Examine the role of educator support and guidance in improving STNs’ RtCP. Research could focus on the impact of different teaching methods and the availability of resources on student outcomes.

Investigate the psychological impact of the COVID-19 pandemic on STNs and the long-term effects on their mental health and professional readiness. This could help in developing targeted mental health support services for nursing students.

## Conclusion

It is evident from the perspective of STNs that the lack of meaningful personal relationships within the CLE has significantly strained interactions, both among STNs and between STNs and their educators. This fragmented and tense environment has hindered effective collaboration and support, resulting in compromised patient care and stunting the professional growth of STNs. Moreover, these challenges have raised concerns about their readiness for clinical practice. The COVID-19 pandemic has further aggravated these issues, underscoring the urgent need for improved guidance and support systems within the CLE to better prepare STNs for their roles. The study effectively explored the readiness of STNs for clinical practice in the post-pandemic era. The findings highlighted both positive and negative perspectives, with adaptation and open-mindedness aiding readiness to practice, while intimidation and strained relationships hindered confidence and autonomy. These insights align with the study’s purpose by revealing the dual impact of the pandemic on STNs’ readiness to practice post-COVID–19 era. The purpose was met as the study provided a comprehensive understanding of the challenges and opportunities faced by STNs. For future improvements, it is suggested that the CLE should focus on fostering meaningful relationships and providing robust support systems to better prepare STNs for their roles.
